# Sociocultural factors associated with persistent prescription opioid use (PPOU) among Puerto Rican adults in Massachusetts

**DOI:** 10.1371/journal.pone.0290104

**Published:** 2023-08-22

**Authors:** Inyene E. Essien-Aleksi, Yuan Zhang, Ainat Koren, Natalia Palacios, Luis M. Falcon, Katherine L. Tucker

**Affiliations:** 1 School of Nursing and Health Sciences, Merrimack College, North Andover, Massachusetts, United States of America; 2 Solomont School of Nursing, University of Massachusetts Lowell, Lowell, Massachusetts, United States of America; 3 Department of Public Health, University of Massachusetts Lowell, Lowell, Massachusetts, United States of America; 4 College of Fine Arts, Humanities & Social Sciences, University of Massachusetts Lowell, Lowell, Massachusetts, United States of America; 5 Department of Biomedical & Nutritional Sciences, University of Massachusetts Lowell, Lowell, Massachusetts, United States of America; Johns Hopkins School of Medicine and Kennedy Krieger Institute, UNITED STATES

## Abstract

**Background:**

Increasing numbers of opioid-overdose deaths have been witnessed among Hispanics and other underserved populations in Massachusetts. Puerto Rican adults (PRs) have a disproportionately higher prevalence of chronic diseases than non-Hispanic White adults—conditions linked to increased prescription opioid use and misuse. Stress indicators, including low acculturation, low social support, and perceived discrimination, have been recognized as correlates of chronic diseases. However, little research has been undertaken on how these socio-cultural factors relate to persistent prescription opioid use among PRs. This study evaluated the prevalence of prescription opioid use and socio-cultural factors associated with persistent prescription opioid use among PRs.

**Methods:**

Data from the prospective population-based Boston Puerto Rican Health Study, at baseline, ~2-year, and ~ 6-year follow-up, were used to estimate prescription opioid use prevalence and its associations with acculturation, social support, and perceived discrimination. Analyses were conducted using multivariable binary logistic regression modeling.

**Results:**

The study sample was comprised of 798 PRs (age 56.5 ± 7.5y) with data at all three-time points. A high prevalence of prescription opioid use was observed and was associated with lower household income. PRs with experiences of perceived discrimination had higher odds of persistent prescription opioid use (y/n; OR = 2.85, 95% CI: 1.46–5.58). No significant associations were found between acculturation, social support, and persistent prescription opioid use.

**Conclusion:**

Our study reported a high prevalence of prescription opioid use in PRs, with persistent prescription opioid use significantly associated with perceived discrimination. Future programs to limit discrimination practices may reduce persistent prescription opioid use and opioid-related complications among PRs.

## Introduction

Prescription opioid use among Americans has declined nationally due to regulatory and public health measures to contain the ongoing opioid crisis [1,2]. Historically, non-Hispanic Whites used more prescription opioids and suffered more from opioid-related complications than other segments of the American population [3]. However, there has been a recent uptick in opioid overdose deaths among underserved communities, including Hispanics [4,5]. Among Hispanics in Massachusetts, opioid-related overdose fatality (OROF) increased approximately by one hundred and twenty percent between 2014 and 2020 [5]. Despite the surge in OROF rates among Hispanics, few studies have explored potential socio-cultural factors that may be driving these deaths, including ecological level factors associated with prescription opioid use (POU) and persistent prescription opioid use (PPOU) among members of this subgroup.

**Fig 1:**
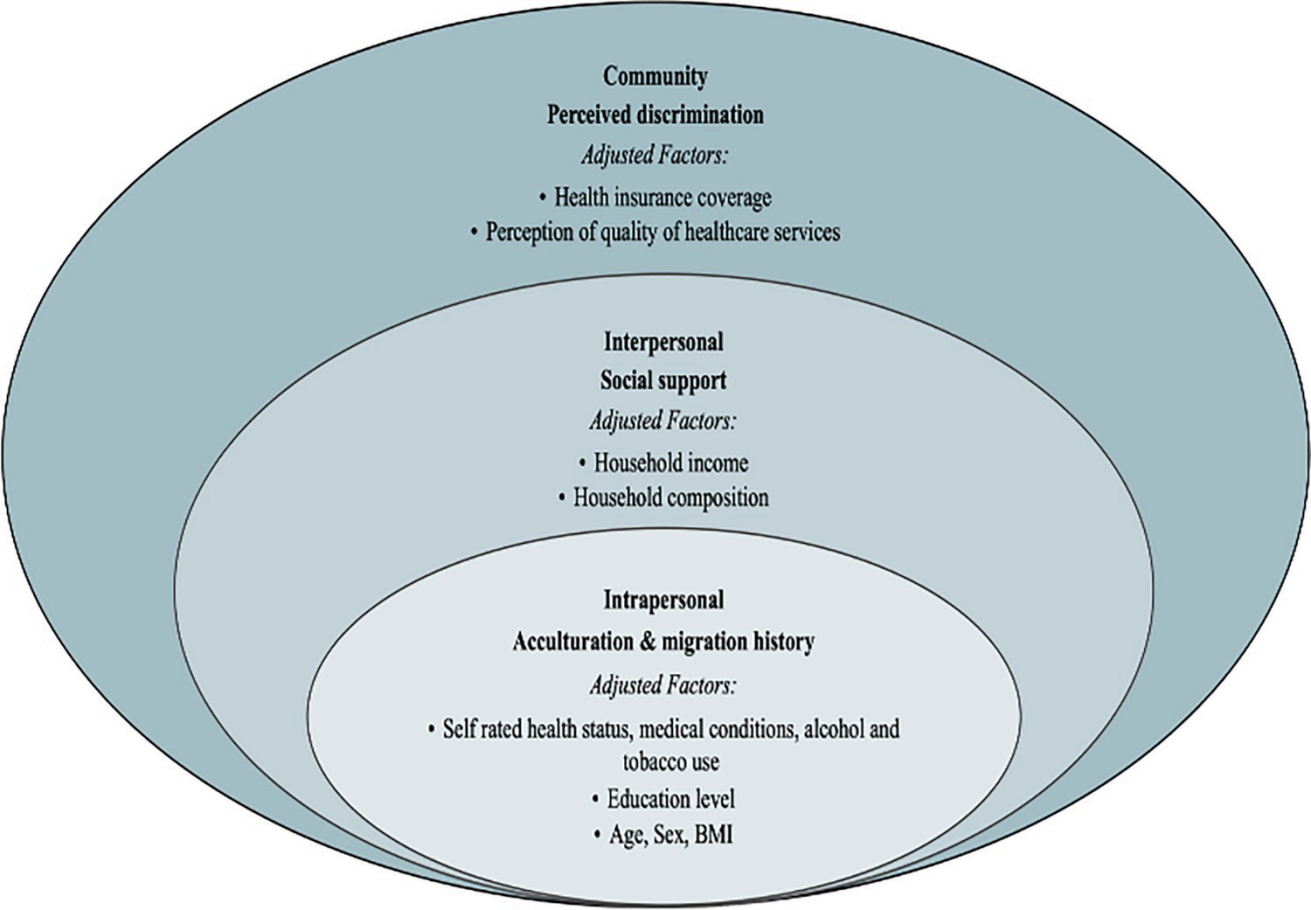
Adapted Socio-Ecological Model framework for the evaluation of PPOU in the Boston Puerto Rican Health Study cohort.

The etiology of opioid misuse and POU-related complications is multifactorial [3]. Currently, literature on causal pathways for chronic diseases has illustrated a linkage between a higher burden of chronic stress (allostatic load) and disparities in adverse health consequences among underserved populations, including Hispanics [6–8]. Hispanics have a higher burden of chronic stress due to socio-environmental challenges, including higher poverty levels, lower educational status, immigration-related problems, acculturation, perceived discrimination, and impaired social support [9–15]. Among specific Hispanic subgroups, Puerto Rican adults (PRs) have disproportionately higher allostatic load and suffer an excessive prevalence of comorbid health conditions, including obesity, hypertension, type 2 diabetes, and osteoarthritis, than other Hispanic groups [11,12,14,16–19]. Predictors of allostatic load, including low acculturation, low social support, and perceived discrimination, have been known to correlate with poorer health outcomes and chronic medical conditions among PRs [13,14,20,21]. Given the established link between stress, allostatic load, and chronic diseases, PRs may be at greater risk of PPOU, an indirect predictor of prescription opioid misuse, prescription opioid-related complications, and OROF in the general population [20–27]. This study adopted the Socio-Ecological Model (SEM) as a theoretical framework to examine the prevalence of POU among PRs and whether differences existed in POU prevalence by demographic factors, including age, gender, household income, and education status (Fig 1). Further, we explored whether acculturation and migration history (intrapersonal), social support and network size (interpersonal), or perceived discrimination (community) predicted PPOU among PRs without cancer-related medical conditions. Examination of PPOU in the context of stress and allostatic load [9,13,14,20,21] could potentially support preventative measures and culturally specific initiatives to reduce PPOU and curb the rising opioid crisis among PRs.

### Theoretical framework

The Ecological Systems Theory by McLeroy [28], now recognized as the Socio-Ecological Model (SEM), is a valuable framework for investigating complex socio-determinant of health (SDOH) that may influence risk behaviors, beliefs, and health outcomes [29,30].The SEM describes five levels of influences that affect health behaviors. These depict the interrelationship between health behaviors and factors within the intrapersonal, interpersonal, institutional (organizational), community, and public policy levels. The overlapping ring of the model assumes that factors within each level interact and influences each other. In this context, a single-level analysis or individual-oriented interventions may not adequately explain a public health issue per the SEM [30]. Exploring potential factors contributing to risk of PPOU among PRs is fundamental to future public health measures to mitigate their risk of prescription opioid-related complications. As such, this study investigated multi-level SEM factors driving PPOU, including intrapersonal- (acculturation and migration history), interpersonal- (social support), and community-level (perceived discrimination) factors among PRs. This multi-level approach provided valuable insights into the socio-environmental conditions of PRs and how these factors may shape their risk of PPOU.

## Methods

### Study design

Data from the Boston Puerto Rican Health Study (BPRHS), a prospective population-based cohort study examining the longitudinal impacts of chronic physiological stress (allostatic load) and health outcomes (such as depression, metabolic diseases, cognitive impairment, and physical disability) among PRs were used for this study [12,14]. This study was approved by the Institutional Review Board (IRB) at the University of Massachusetts Lowell (IRB exempt # 21–199). Participants in the main study provided written informed consent, which was obtained by trained bilingual interviewers. This study used de-identified secondary data and was exempt from collecting additional informed consent.

### Participants and setting

The BPRHS cohort of PRs (aged 45–75 years) were recruited from the Greater Metropolitan Boston area between 2004–2009. Recruitment was performed using door-to-door enumeration and community outreach approaches. The 2000 US Census data was used to identify census tracks with at least 25 PRs, with the age range of 45–75 years old. Within the selected census blocks, households were visited at least three to six times per week, until a response was obtained, and one qualified individual per household was randomly selected for interviews at their respective homes [31]. Excluded participants included: those with a low Mini-Mental State Examination (MMSE) score (≤ 10), those unable to answer questions due to severe health conditions, and those with plans to relocate from the Greater Boston area within two years [12]. The total recruited sample included 1502 participants at baseline, 1258 at ~2-year follow-up, and 961 at ~6-year follow-up. Eligible participants for this analysis were PRs who completed surveys consecutively on their prescription opioid use at baseline, 2-year, and 6-year follow-up and reported no cancer-related conditions (n = 798). Further detailed description of the BPRHS cohort was published elsewhere [12].

### Variables

#### Outcome variables

*Persistent prescription opioid use (PPOU)*. PPOU was defined as participants with prescription opioid use at least two consecutive times out of the three points: at baseline, 2- years, and 6-year follow-up. Non-consecutive prescription opioid users were defined as participants not using prescribed opioids continuously over the three-time points. Non-consecutive prescription opioid users and non-users (no self-reported opioid use) were categorized as non-users (reference group). We pooled participants on prescribed short-term opioids (non-consecutively) and non-users to compare the differences between persistent prescription opioid users (those at higher risk for opioid-related complications) versus the reference group (lower risk or non-users). Of note, the BPRHS measured prescription opioid use with a single item, denoting 0 as “no opioid use” and 1 as “yes to opioid use,” and confirmed by observation of prescription containers. The measured construct did not account for opioid drug type prescribed, strength, quantity, data dispensed, indication for use, or duration of therapy.

*Prevalence of prescription opioid use*. Prevalence of prescription opioid use was estimated as the total number of PRs without cancer-related medical conditions who reported using prescription opioids at baseline, 2-year, or 6 years over the sum of the specified cohort at baseline, 2-year, and 6-year.

#### Predictor variables

*Acculturation and migration history (Intrapersonal level)*: Baseline acculturation was measured using an adaptation of the Bidimensional Acculturation Scale for Hispanics by Marin and Gamba (1996) [12] during home interviews. The acculturation scale assesses participant’s English language use, psychological acculturation, and length of residence in the US mainland (years in the US) [12,17]. The length of residence in the US was measured as a continuous variable. A language acculturation score of 100% represented complete acculturation (fluency in the English language), whereas 0% represented non-acculturation (fluency in the Spanish language only) [12,32]. A higher psychological acculturation score reflected a greater affinity to Hispanic culture and beliefs. The Bidimensional Acculturation Scale for Hispanics by Marin and Gamba (1996) has demonstrated good internal consistency of 0.97 (Cronbach’s alpha) and is particularly useful in measuring the effects of Hispanic culture and beliefs on their health outcomes [12,13,17,33,34].

*Social support (Interpersonal level)*: Baseline social support was measured with the Norbeck Social Support Questionnaire (NSSQ) [35] during home interviews. The NSSQ measures two dimensions of social support (emotional and tangible social support), network size and relationships in the network [35]. The NSSQ scale asks respondents to identify 16 critical persons providing direct support and essential to their social network [35]. Responses are rated on a 5-point Likert scale: 0, denoting “not at all” and 4 indicating “a great deal” of social support [12,21]. The summation of NSSQ scores reflects the quantity of support experienced by the respondent, which may vary based on network size and the stability of the relationship in the social network [36]. Split-half reliability of the NSSQ subscales has shown a high correlation of items (Affect at 0.97, Affirmation, 0.96, and Aid, 0.89), and good concurrent validity has also been established for the scale [37,38]. Due to the high intercorrelation of items in the NSSQ subscales, the average scores of each dimension of social support were used to evaluate the effects of social support on PPOU [9,36].

*Perceived discrimination (community level)*: Baseline perceived discrimination was measured using a modified version of the Everyday Experiences of Discrimination Scale, during home interviews [20]. This scale contains four items on participants’ experiences of discrimination due to race, ethnicity, and spoken language. Responses were denoted as 1 being "yes" and 0 being "no." Affirmative responses were followed by three questions examining whether participants’ experiences of discrimination occurred in a healthcare establishment, how it happened, and whether these discriminatory experiences interfered with the ability to access healthcare services [12]. The perceived discrimination scale does not specify the timeframe of discrimination experienced [20]. Internal consistency for the scale has ranged from 0.77 to 0.87 (Cronbach’s alpha) [7,20], and further large-scale studies have demonstrated good construct and convergent validity of the perceived discrimination scale across multiethnic populations [39–41].

#### Covariates

This study adjusted for several factors at each level of the modified SEM framework (Fig 1), measured during home interviews at baseline (2004–2009). These factors included age, gender, education, alcohol use, tobacco use, comorbidities, BMI, and self-rated health status at the intrapersonal level; total household income and household composition at the intrapersonal level; and health insurance coverage and participants’ perception of the quality of healthcare services at the community level. Gender was categorized as female vs. male. Education was categorized as less than/equal to 12^th^ grade vs. some college or higher (some college). Medical comorbidities were categorized based on the participant’s number of current medical conditions (0 vs. ≥ 1). Self-rated health status was measured as excellent, very good, good, fair, and poor. Alcohol use was calculated as the frequency of alcohol use by the respondents, categorized as none, moderate or heavy use in the past year. Smoking status was categorized as never (less than 100 cigarettes in entire life), smoked in the past but not currently, or currently smoking. The household composition was categorized as 1, 2, 3, or ≥ 4 based on the number of persons in the participant’s household. Participants’ perception of the quality of healthcare service was categorized as very satisfied, satisfied, somewhat satisfied, or not at all satisfied. Age, BMI, and total household income were measured as continuous variables.

### Data analysis

Data analyses were conducted using SAS version 9.4 (SAS Institute, Cary, NC). Baseline, 2-year, and 6-year datasets were merged across timepoints, excluding participants with self-reported cancer-related medical conditions. Descriptive analyses were conducted for all SEM variables, and parameters reported as mean ± standard deviations (SD) for continuous variables and percentage (%) for categorical variables. Prevalence was computed per 100 persons with 95% confidence intervals (CI) and stratified by age, gender, household income, and education categories. Associations between variables at each level of the SEM model and PPOU were tested using cross-tabulation analysis or independent samples t-test. Multivariable binary logistic regression models were used to test whether baseline acculturation and migration history, social support and network size and perceived discrimination were independently associated with PPOU (Tables 1 and 2). Covariates significantly associated with PPOU at the bivariate level (*P* < 0.05) were adjusted in these multivariate models.

**Table 1:** Baseline sample characteristics and socio-cultural measures of the study cohort (categorical variables) by persistent prescription opioid use (n = 798).

Characteristics (*Categorical variables*)	All participants (n = 798) (%)	Persistent prescription opioid users (n = 52) (%)^a^	Non- users (n = 746) (%)^a^
**Intrapersonal level**			
Women	72.9	69.3	73.1
Education (≤ 12^th^ grade)	85.2	94.2	84.5
Alcohol consumption			
Heavy	6.3	7.8	6.2
Moderate	37.7	23.5	38.7
None	56.0	68.6	55.1
Current smoker (yes)	22.6	**36.5** *	**21.6** *
Self-rated health status (fair & poor)	70.9	**88.5** **	**69.8** **
Comorbidities			
0	3.4	**0.0** ***	**3.6** ***
≥ 1	96.6	**100.0** ***	**96.4** ***
**Interpersonal level**			
Household composition			
1—person	35.0	34.6	35.0
2—people	31.7	40.4	31.1
3—people	15.7	19.2	15.4
≥ 4 people	17.6	5.8	18.4
**Community level**			
Perceived discrimination (yes)	38.3	58.3**	**36.** 8**
Health Insurance coverage (yes)	95.6	3.9	4.4
Perception of quality of healthcare			
Very satisfied	42.1	**40.4** *	**42.2** *
Satisfied	39.9	**26.9** *	**40.8** *
Somewhat and not at all satisfied	18.0	**32.7** *	**17.0** *

Crosstabulation results of Chi-squared test was performed for categorical SEM variables to compare the percentage differences of persistent prescription opioid users versus non-users.

^a^ *P* < .05

*, *P* < .01

**, *P* < .001

***, bold values are significant at 95% level.

**Table 2:** Baseline sample characteristics and socio-cultural measures of the study cohort (continuous variables) by persistent prescription opioid use (n = 798).

Characteristics (*Continuous variables*)	All participants (n = 798) Mean ± SD	Persistent prescription opioid users (n = 52) mean ± SD ^a^	Non- users (n = 746) mean ± SD ^a^
**Intrapersonal level**			
Age	56.5 ± 7.5	55.2±7.5	56.7 ± 7.4
BMI	32.5 ± 6.9	33.6 ± 9.0	32.4 ± 6.7
Acculturation			
Length of residence	33.8 ± 11.9	33.5 ± 11.2	33.8 ± 11.9
Language acculturation	23.6 ± 21.8	22.2 ± 20.5	23.6 ± 21.9
Psychological acculturation	18.2 ± 6.76	17.5 ± 7.2	18.2 ± 6.7
**Interpersonal level**			
Social support			
Network size	5.4 ± 3.2	4.7 ± 2.6	5.4 ± 3.3
Average emotional support	14.1 ± 3.0	13.6 ± 2.8	14.1 ± 3.0
Average tangible (aid) support	6.0 ± 2.0	5.6 ± 2.1	5.9 ± 2.0
Household Income	$17664 ± $17611	**$12215 ± $8194** ***	**$18047 ± $18032** ***

Independent samples t-test results comparing the mean differences of SEM variables between persistent prescription opioid users versus non-users. *SD* standard deviation, *BMI* body mass index.

^a^ *P* < .001

***, bold values are significant at 95% level.

## Results

### Participants characteristics

The study consisted of 798 participants (72.9% women) with a mean age of 56.5 years at baseline. Overall, 52 (6.5%) of PRs used prescription opioids persistently compared to 746 (93.5%) non-users (Table 1). Persistent prescription opioid users reported lower educational status, lower use of alcohol, lower household income, current smoking status, multiple comorbidities, poorer self-rated health status, more experiences of perceived discrimination, and lower satisfaction with the quality of healthcare services.

#### Prevalence of prescription opioid use by demographic factors

The prevalence of POU among the cohort was 11.5 per 100 persons (95% CI: 9.1–13.8) at baseline, 10.3 per 100 persons (95% CI: 8.1–12.5) at 2-year, and 14.6 per 100 persons (95% CI:11.9–17.2) at 6-year follow-up. The prevalence of POU varied by age categories in the 6-year cohort (*P* = 0.02), with younger participants (< 61.3 years) having a higher prevalence of POU (*P* = 0.02), but not at baseline or 2-year. Additionally, a higher prevalence of POU was associated with lower household income at baseline (*P* = 0.0001), 2-year (*P* = 0.0001), and 6-year (*P* = 0.02).

#### Sociocultural factors associated with persistent prescription opioid use

Univariate modeling of PPOU by baseline primary SEM factors showed no significant associations with language acculturation, psychological acculturation, length of residence in the U.S. mainland, social support network size, emotional social support, and tangible (aid) support. However, perceived discrimination (OR = 2.41, 95% CI: 1.32–4.37) was associated with higher odds of PPOU. Multivariable logistic regression revealed greater odds of PPOU among PRs experiencing perceived discrimination (OR = 2.85, 95% CI 1.46–5.58), after adjusting for smoking status, self-rated health status, comorbidities, household income, and perception of healthcare quality. There were no significant associations between PPOU and language acculturation, psychological acculturation, social support network size, average aid, or emotional social support (Table 3). C-statistics supported the model’s goodness of fit (0.81). PRs who reported experiences of perceived discrimination were 185% times more likely to use prescription opioids persistently than those without experiences of perceived discrimination.

**Table 3:** Multivariate logistic regression models for acculturation, social support, perceived, and persistent prescription opioid use among the Boston Puerto Rican Cohort.

	Model 1	Model 2	Model 3	Model 4	Model 5
Variables	OR (95% CI) ^a^	OR (95% CI) ^a^	OR (95% CI) ^a^	OR (95% CI) ^a^	OR (95% CI) ^a^
**Language acculturation**	0.98 (0.98–1.02)	-	-	0.99 (0.97–1.01)	1.00 (0.98–1.02)
**Psychological acculturation**	0.99 (0.94–1.04)	-	-	1.00 (0.94–1.05)	0.98 (0.92–1.04)
**Length of residence in US mainland**	0.99 (0.98–1.02)	-	-	1.00 (0.97–1.03)	0.98 (0.96–1.02)
**Network size**	-	0.93 (0.83–1.02)	-	0.91 (0.81–1.01)	0.94 (0.83–1.06)
**Average emotional support**	-	0.97 (0.86–1.09)	-	1.00 (0.88–1.12)	1.04 (0.91–1.19)
**Average tangible (aid) support**	-	0.94 (0.80–1.12)	-	0.92 (0.76–1.10)	0.92 (0.75–1.12)
**Perceived discrimination (yes)**	-	-	**2.41** (1.33–4.37)*	**2.67** (1.43–4.98)*	**2.85** (1.46–5.58)*

Model 1: Acculturation and migration history (intrapersonal level), unadjusted for covariates.

Model 2: Social support (interpersonal level), unadjusted for covariates.

Model 3: Perceived discrimination (community level), unadjusted for covariates.

Model 4: All three-level SEM primary factors, unadjusted for covariates.

Model 5: All three-level SEM primary factors, adjusted for smoking, comorbidities, self-rated health status, household income, and perception of quality of healthcare services.

Note:—indicates SEM variables not evaluated in the model, and perceived discrimination variable reference group is no. Baseline language acculturation was treated as a continuous variable, summation of scores (ranging 0–100), with lower scores indicative of more affinity to Spanish language and higher scores indicating more affinity to English language-based acculturation. Baseline psychological acculturation was treated as a continuous variable, summation of scores (ranging from 0–50) with lower scores indicative of more affinity to Puerto Rican culture and higher scores indicative of more U.S. psychological-based acculturation. Length of residence in the U. S. was assessed as a continuous variable, summation of scores (ranging from 0–58 years). Network size was treated as a continuous variable, summation of scores (ranging from 0–16), with higher scores indicating greater number of persons in the social support network. Average emotional support was treated as a continuous variable, summation of scores (ranging from 3.5–33.7), higher scores indicating increased emotional support. Average tangible (aid) support was treated as a continuous variable, summation of scores (ranging from 0–16), with higher scores indicating increased tangible (aid) support.

^a^ *OR =* odds ratio, *95% CI* = confidence intervals range lower to upper limit with *P* < 0.05 ^*^, bold values significant at 95% level.

## Discussion

Among a sample of Puerto Rican adults without cancer-related medical conditions in Greater Boston, the prevalence of POU increased over time, with a prevalence of persistent prescription opioid users of 6.5%. Experiences of perceived discrimination (community-level) at baseline were associated with PPOU. No significant association was observed between acculturation, migration history, social support, and PPOU. This is the first study to examine POU patterns and SEM predictors of PPOU in a large sample of PRs. The reported prevalence of POU among PRs was comparable to estimates in the general population [42,43]. The prevalence of long-term POU has ranged from 1.3% to 25% for adults with chronic non-cancer pain [44] and as high as 28.6% for adults with arthritis [45], and was notably higher among patients with adverse health status and more chronic medical comorbidities [26,45–47].

An important finding of this study is that perceived discrimination (community-level) was associated with PPOU. Based on previous studies, the underlying belief has been that experiences of racial bias and discrimination decrease exposure to prescription opioid among minority groups, including Hispanics [48–50]. In contrast, our study observed a positive correlation between the experiences of perceived discrimination and PPOU among PRs. Several studies among Hispanics have linked discrimination to greater substance use disorders (alcohol and opioid drug misuse) and poor health outcomes [20,21,51–53]. The reason for the higher prevalence of PPOU among PRs experiencing discrimination is unclear. However, a possible explanation is that PRs experiencing discrimination may seek relief by using more substances. It is also important to note that most PRs in Massachusetts, as U.S. citizens, have access to health care through MassHealth, whereas larger proportions of other Hispanic groups are uninsured. It is also possible that reverse causality contributes to this relationship, where individuals with PPOU may feel perceived discrimination due to cognitive biases related to opioid use, but scant evidence exists to support this hypothesis. Nevertheless, given the diversity within the Hispanic population, further studies should examine whether group-specific factors, such as culture, genetics, and sociodemographic status, may influence determinants of PPOU among PRs.

In the study, we evaluated social and cultural determinants of PPOU, with variables such as perceived discrimination, which is a known contributor to allostatic load [20,21]. Therefore, a potential explanation for the association between perceived discrimination and PPOU may be the allostatic load hypothesis [54–56], given the high rates of chronic medical conditions and poor health status documented among PRs [12]. Chronic exposure to stress can alter normal physiological stress response processes, leading to allostatic overload [6,20]. It is possible that exposure to stressors related to discrimination could have altered stress response processes among PRs over time, leading to their increased risk of chronic medical conditions and PPOU. This assumption has been supported by previous studies linking higher allostatic load scores and risk of risky behaviors, such as alcohol use and smoking, among PRs experiencing perceived discrimination [20,21]. As these risk profiles (such as smoking, alcohol use, and greater numbers of chronic medical conditions) are associated with PPOU [22–25], determinants of allostatic overload may be the conduit through which the association between perceived discrimination and PPOU can be explained.

Numerous studies have also linked prescription opioid use to patients with lower income status [47,57–59]. For example, higher-income transgender patients have reported fewer discrimination experiences than those with lower-income [60]. Factors such as stigmatizing attitudes, childhood abuse, and neglect have been identified as correlates of discrimination and healthcare abuse among patients with long-term opioid use and OUD [61–63]. As such, other contributing factors may also explain the relationship between perceived discrimination and PPOU.

This study also explored whether acculturation, migration history (intrapersonal), social support, and network size (interpersonal) predicted PPOU among PRs without cancer-related medical conditions. Our findings indicated no associations between SEM factors of acculturation and migration history (intrapersonal) or social support (interpersonal) with PPOU. These findings were unexpected, as lower acculturation and social support have been linked to an increased burden of chronic medical conditions among PRs [9,13]. Further, the findings were inconsistent with a retrospective study of Hispanics, which found greater English language acculturation associated with lower odds of prescription opioid use [64]. In that study, nativity status on the U.S. mainland was significantly associated with increased odds of prescription opioid use [64]. Perez et al.’s [64] study was conducted with a large sample of Hispanic adults, including a broader age range (18–74 years) and predominantly Mexican descent (29.6%) living in Chicago, Miami, the Bronx, and San Diego. Therefore, it is possible that our smaller sample size, age differences, and locality (Greater Boston) impacted our participant’s cultural perspectives, as well as measures of acculturation. In addition, other comparable studies have reported a mixed relationship between the construct of acculturation and various health outcomes among Hispanics [65–67]. For example, greater acculturation has long been considered a risk factor for substance misuse [53,66–69]; but more recent studies have contradicted this assessment [64,68].

In the present study, participants with lower household income levels were likelier to have a higher prevalence of POU and PPOU. In many cross-sectional and longitudinal studies, lower-income status has been linked to prescription opioid use [47,57–59,64]. One explanation for this association could be the increased burden of health disparities and socioeconomic inequities among persons of lower economic standing [70]. Therefore, PRs with disparities in chronic diseases and lower household income could be mirroring a general trend in prescription opioid use risks.

The prevalence of POU was higher among participants younger vs. older than 61.3 years at the 6-year follow-up. Prevalence of POU and PPOU were not consistently associated with age, gender, and education status across the rest of the cohort. In a systemic review evaluating predictors of long-term prescription opioid use, younger age was associated with long-term prescription opioid use [44]. However, research has not consistently linked younger age and long-term use of prescription opioids or PPOU [26,42,46,47]. For example, Mojtabai [26] demonstrated that older adults with public health insurance had higher long-term prescription opioid use than their younger counterparts. This increase in PPOU among older adults could have been partly due to gains in access to public health insurance (e.g., Medicare) with advancing age and access to care [45,47]. Finally, PRs who used opioids persistently had several comorbid medical and psychiatric conditions, such as anxiety disorder, arthritis, respiratory illness, and stomach and intestinal disorders. Similar associations between short-term and long-term prescription opioid use have been reported with conditions such as post-traumatic stress disorder, mental illness, osteoarthritis, back pain, neuropathic pain, and HIV/AIDs among U.S. adults [24,71–74].

### Strengths and limitations

The present study has several key strengths. To our knowledge, this is the first longitudinal cohort study to examine the prevalence of prescription opioid use and sociocultural factors associated with PPOU among PRs with an ecological framework. This study explored a range of SEM factors, and adapting the SEM theoretical framework allowed for an in-depth analysis of multi-level socio-determinant health factors associated with PPOU. Although this analysis provides insight into the sociocultural factors associated with PPOU among PRs, the study has several limitations. Our measured construct of PPOU was limited. Self-reported indicators of POU were used to estimate the probability of long-term prescription opioid use in the studied sample. The measure of PPOU was constructed as two consecutive exposures to prescribed opioids, which may not have captured a representative definition of PPOU in the clinical setting. However, as there is no standardized definition of PPOU [75–78], we used this inclusion criterion to ensure that participants were long-term opioid users and not using opioids only briefly. Additionally, the measure of POU did not include medication information, such as dose, frequency, duration, date prescribed, date dispensed, refilled frequency, pain status, and indication for POU. Our analyses were based on a previously collected longitudinal data set; therefore, other correlates and confounders of POU, PPOU, and perceived discrimination, such as perception of prescription opioids, history of ADHD, and other illicit substance use, et al., were not available for inclusion in this analysis [46,79,80]. Depression was not examined as a separate confounding variable but assessed as part of the medical comorbidities variable. In addition, as we used self-reported secondary data, we could not examine whether participants were taken off prescription opioids or had prescription opioid misuse at follow-up time points. Finally, the majority of our sample were women (73%), which may limit the precision of the overall estimates in relation to the general population of PRs in this age group.

### Conclusions

In summary, the prevalence of POU among PRs with non-cancer-related medical conditions increased over time. Participants with lower household incomes were more likely to engage in POU. Based on the adapted ecological framework, PRs who reported experiences of perceived racial discrimination were likelier to use prescription opioids persistently than those without experiences of perceived discrimination. Acculturation, migration history, and social support were not significantly associated with PPOU. Our study findings have implications for healthcare practitioners caring for patients with opioid-related complications. Addressing modifiable factors, such as interventions to reduce discriminatory practices, may impact PPOU exposure and reduce opioid-related complications among PRs. Educational programs targeting healthcare professionals could be initiated to promote changes in practice and enhance their knowledge of the health implications of discrimination among vulnerable populations like PR adults. This study highlights the need to explore whether healthcare initiatives aiming to promote equity, diversity and combat discrimination could potentially reduce persistent prescription opioid use and opioid-related complications among PRs. Further research should explore other social and cultural variables that may influence the relationship between perceived discrimination and PPOU among PRs and other high-risk groups.
